# F146. S-KETAMINE-INDUCED NMDA RECEPTOR BLOCKADE DURING NATURAL SPEECH PRODUCTION AND ITS IMPLICATIONS FOR FORMAL THOUGHT DISORDER IN SCHIZOPHRENIA: A PHARMACO-FMRI STUDY

**DOI:** 10.1093/schbul/sby017.677

**Published:** 2018-04-01

**Authors:** Tilo Kircher, Arne Nagels

**Affiliations:** Marburg University

## Abstract

**Background:**

FTD is a dimensional, phenomenologically defined construct, which can clinically be subdivided into positive (pFTD) versus negative (nFTD) as well as objective versus subjective symptom clusters. Structural and functional changes in the lateral temporal language areas have been related to formal thought disorder (FTD) in schizophrenia. Continuous, natural speech production activates the right lateral temporal lobe in schizophrenia, as opposed to the left in healthy subjects. Positive and negative FTD can be elicited in healthy subjects by glutamatergic NMDA blockade with ketamine. It is unclear, whether the glutamate system is related to the reversed hemispheric lateralization during speaking in patients.

**Methods:**

In a double-blind, cross over, placebo-controlled study, 15 healthy, male, right-handed volunteers overtly described 7 pictures for 3 minutes each, while BOLD signal changes were acquired with fMRI. As a measure of linguistic demand, the number of words within 20-second epochs was correlated with BOLD responses.

**Results:**

Participants developed S-ketamine-induced psychotic symptoms, particularly positive FTD. Ketamine vs. placebo was associated with enhanced neural responses in the right middle and inferior temporal gyri.

**Discussion:**

Similar to a previous fMRI study in schizophrenia patients vs. healthy controls applying the same design, S-ketamine reversed functional lateralization during speech production in healthy subjects. Results demonstrate an association between glutamatergic imbalance, dysactivations in lateral temporal brain areas, and FTD symptom formation. Left superior temporal gyrus (STG) cortical volume is decreased in schizophrenia patients (SZ) with pFTD in structural magnetic resonance imaging (sMRI) studies and shows reversed activation in functional MRI (fMRI) experiments during speech production. pFTDs are related to synaptic rarefication in the glutamate system of the superior and middle lateral temporal cortices.

**References:**

1. Nagels A, Cabanis M, Oppel A, Kirner-Veselinovic A, Schales C, Kircher T. Neuropsychopharmacology. 2017 Nov 6. doi: 10.1038/npp.2017.270.

2. Kircher T, Liddle PF, Brammer MJ, Williams SC, Murray RM, McGuire PK. Reversed lateralization of temporal activation during speech production in thought disordered patients with schizophrenia. Psychol Med. 2002 Apr;32(3):439–49.

3. Kircher T, Bröhl H, Meier F, Engelen J. Formal Thought Disorders (FTD): From Phenomenology to Neurobiology. Lancet Psychiatry, in press.

